# An Update on the Therapeutic Potential of Antimicrobial Peptides against *Acinetobacter baumannii* Infections

**DOI:** 10.3390/ph16091281

**Published:** 2023-09-11

**Authors:** Karyne Rangel, Guilherme Curty Lechuga, David W. Provance, Carlos M. Morel, Salvatore G. De Simone

**Affiliations:** 1 Center for Technological Development in Health (CDTS), National Institute of Science and Technology for Innovation in Neglected Population Diseases (INCT-IDPN), Oswaldo Cruz Institut, Oswaldo Cruz Foundation, Rio de Janeiro 21040-900, RJ, Brazil; karyne.rangelk@gmail.com (K.R.); gclechuga@gmail.com (G.C.L.); bill.provance@fiocruz.br (D.W.P.J.); carlos.morel@fiocruz.br (C.M.M.); 2 Epidemiology and Molecular Systematics Laboratory (LEMS), Oswaldo Cruz Institut, Oswaldo Cruz Foundation, Rio de Janeiro 21040-900, RJ, Brazil; 3 Program of Post-Graduation on Science and Biotechnology, Department of Molecular and Cellular Biology, Biology Institute, Federal Fluminense University, Niterói 22040-036, RJ, Brazil; 4 Program of Post-Graduation on Parasitic Biology, Oswaldo Cruz Institute, Oswaldo Cruz Foundation, Rio de Janeiro 21040-900, RJ, Brazil

**Keywords:** AMP, *Acinetobacter baumannii*, resistance, mechanism of action, mechanism of action

## Abstract

The rise in antibiotic-resistant strains of clinically important pathogens is a major threat to global health. The World Health Organization (WHO) has recognized the urgent need to develop alternative treatments to address the growing list of priority pathogens. Antimicrobial peptides (AMPs) rank among the suggested options with proven activity and high potential to be developed into effective drugs. Many AMPs are naturally produced by living organisms protecting the host against pathogens as a part of their innate immunity. Mechanisms associated with AMP actions include cell membrane disruption, cell wall weakening, protein synthesis inhibition, and interference in nucleic acid dynamics, inducing apoptosis and necrosis. *Acinetobacter baumannii* is a critical pathogen, as severe clinical implications have developed from isolates resistant to current antibiotic treatments and conventional control procedures, such as UV light, disinfectants, and drying. Here, we review the natural AMPs representing primary candidates for new anti-*A. baumannii* drugs in post-antibiotic-era and present computational tools to develop the next generation of AMPs with greater microbicidal activity and reduced toxicity.

## 1. Introduction

The rise of antibiotic resistance is a major aid to global mortality statistics and represents a challenge for societies, including healthcare providers, governmental agencies, and the pharmaceutical industry. The inability to develop new antibiotics to interfere with drug-resistant pathogens suggests the world is heading toward a post-antibiotic era [1,2]. For bacteria, three types of antimicrobial resistance have been described: intrinsic, acquired, and adaptive; the last is known as resistance due to changes in bacterial phenotype [3,4,5,6,7,8,9,10,11]. The main mechanisms of antimicrobial resistance are target modification or mutation, efflux pumps, permeability reduction, hydrolysis or enzymatic inactivation, metabolic enhancement or auxotrophy, community cooperative resistance, target protective protein (TPPs), changes in cell morphology, and self-repair systems (Table 1). While many mechanisms lead to resistance, the exposure of microbes to inadequate doses of antimicrobial drugs can trigger their evolution, contributing to the selection of antimicrobial resistance [12,13].

According to Magiorakos et al. (2012), a multidrug-resistant (MDR) strain shows resistance to at least one antimicrobial in more than three classes of antimicrobials; an extensively drug-resistant (XDR) strain displays resistance to at least one antimicrobial among all classes of antimicrobials, while a pan drug-resistant (PDR) strain is resistant to all antimicrobial agents [14]. In hospital settings, ESKAPE pathogens (*Enterococcus faecium*, *Staphylococcus aureus*, *Klebsiella pneumoniae*, *A. baumannii*, *Pseudomonas aeruginosa*, and *Enterobacter species*) comprise the main opportunistic pathogens in nosocomial infections, posing a global health threat due to their ability to evade traditional antibiotics used in conventional therapies, accounting for increased morbidity and mortality in healthcare systems [15,16,17].

*A. baumannii* has long been associated with human disease [18] and has globally emerged as a concerning hospital-related pathogen, frequently presenting MDR, XDR, and PDR phenotypes. The *Acinetobacter calcoaceticus–A. baumannii* complex (Acb) belongs to the *Moraxellaceae* family [19], comprising the following species: *A. calcoaceticus*, *A. baumannii*, *A. pittii*, *A. nosocomialis*, *A. seifertii*, and *A. lactucae* (a later heterotypic synonym of *A. dijkshoorniae*) [20,21]. Acb species differ in epidemiology, pathogenicity, and antimicrobial resistance [22]. While their genetic and physiological relatedness makes them difficult to distinguish phenotypically using standard laboratory methods [23], *A. baumannii* is the most widespread in hospitals, causing wounds, skin and urinary tracts infections, and also diseases such as pneumonia, meningitis, and bacteremia [24,25]. All contribute to longer hospital stays, higher treatment costs, and increased morbidity and mortality risks [26].

Treatment options have proven limited for *A. baumannii* due to its extended virolome and resistome, evasion of host immune effectors, survival under extreme environmental conditions, growth in biofilms, and latent growth on a minimal metabolic rate [27,28].

**Table 1:** Bacterial resistance mechanisms against antibiotics.

Antibiotic Resistance Mechanism	Characteristics	Example	Ref.
Target modification or mutation	Mutation or modification of bacterial site will interfere with target matching, thus affecting the effect of antibiotics	Modifying PBPs in MRSA, production of β-lactamases or carbapenemases in genus *Klebsiella*; fluoroquinolone-resistant *S. aureus* *Mycobacterium tuberculosis* resistance to rifampicin is mainly caused by the mutation of the *rpoB* gene and vancomycin-resistant *Enterococcus* (VRE)	[29]
Reduced permeability	Deletion or damage of Omps is a source of bacterial resistance	Loss of porin D2 from outer cell wall in imipenem-resistant *P. aeruginosa*	[30]
Inactivating enzymes	Inactivating enzymes produced by bacteria, such as antibiotic hydrolases or similar enzymes, can hydrolyze or modify antibiotics inside the cell, rendering their inactivation before reaching the target site	Production of penicillin-inactivating β-lactamase by penicillin-resistant *S. aureus*, *Haemophilus influenzae*, and *Escherichia coli* bacteria, gentamicin-resistant *enterococci* via enzymatic inactivation of aminoglycosides and carbapenem-producing Enterobacteriaceae	[31]
Efflux pumps	Pumping of harmful molecules out of the bacterial cell	Increased efflux of tetracycline, macrolides, clindamycin, or fluoroquinolones in *S. aureus*	[32]
Metabolic enhancement or auxotrophy	Core genome mutations change metabolic pathways and induce antibiotic resistance	The genome of clinically pathogenic *E. coli*	[33,34]
Community cooperative resistance	Most bacteria coexist in communities, collectively resisting antibiotic effects; bacterial biofilms are efficiently protective of biofilm-forming bacterial species	*P. aeruginosa*, *S. aureus*, *S maltophilia*, and other bacteria	[35]
Target protective proteins (TPPs)	Bacterial synthetic protein protects antibiotic targets from antibiotics, eliminating their bacteriostatic effects	Clinically isolated *S.aureus* and other *staphylococcus* resistance to fusidic acid due to the level acquisition of genes encoding the FusB-type protein	[36]
Cell morphology changes	Modulating the body’s relative area via absorption efficiency changes can lead to the dilution of antibiotics entering the bacterial cell	Cells of the commonly used model organism *Caulobacter crescentus*	[37]
Self-repair systems	The multiple antibiotic resistance operon of enteric bacteria manipulates DNA repair and outer membrane integrity, enhancing antibiotic resistance	*E. coli* multiple antibiotic resistance (*mar*) loci was recognized as a determinant for cross-resistance to tetracyclines, quinolones, and β-lactams	[38]

AR, antibiotic resistance; Ref., reference; MLSB = macrolide, lincoside, streptogramin; PB, penicillin-binding.

Colistin is, currently, the main therapeutic option against resistant strains of *A. baumannii.* Unfortunately, since its reintroduction, reports on *A. baumannii* colistin resistance mechanisms have been reported, including the complete loss of LPS, modifications of the LPS target or plasmid-encoded MCR genes, and colistin efflux from the cell [39].

The World Health Organization (WHO) recently highlighted the resistance of *A. baumannii* to carbapenems (CRAb) [40,41], which classifies the species as a “priority for research and development of new antibiotic treatments.” CRAb is a “critical” pathogen [42]. Antimicrobial peptides (AMPs) have a high potential for use in the research and development of anti-*Acinetobacter* drugs [43,44].

**Figure 1:**
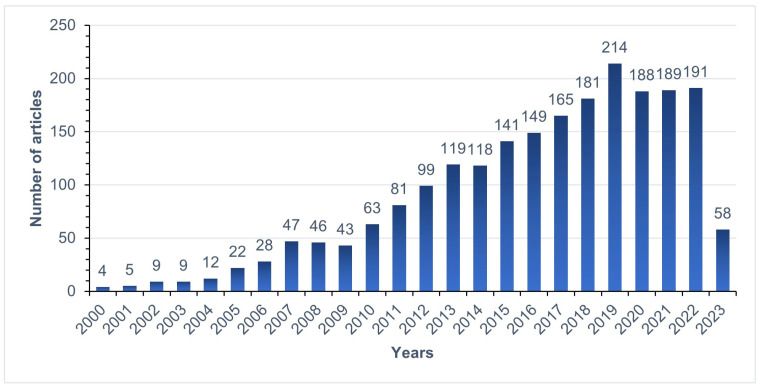
Number of articles selected according to the year of publication.

In this review, from January 2000 to April 2023, an extensive literature search was carried out at PubMed to update current knowledge about the activity of antimicrobial peptides (AMPs), combining keywords related to *Acinetobacter baumannii* and antimicrobial peptides (Figure 1) and finding several AMPs capable of acting against MDR *A. baumannii*. According to our search criteria, no previous publication on this topic was found.

## 2. Antimicrobial Peptides

Antimicrobial peptides, also known as host defense peptides, are produced naturally by living organisms as a part of their innate immune system against pathogens. AMPs are amphipathic molecules of varying molecular weights containing 11–50 amino acids with an overall positive electric charge [45,46], classified as α-helical, β-sheet, or extended peptides [47,48,49]. AMPs are essential in regulating immune processes such as inflammation, activating and recruiting immune system cells [45]. In addition, they can inhibit protein and nucleic acid synthesis, occasionally leading to apoptosis and necrosis [50,51].

**Figure 2:**
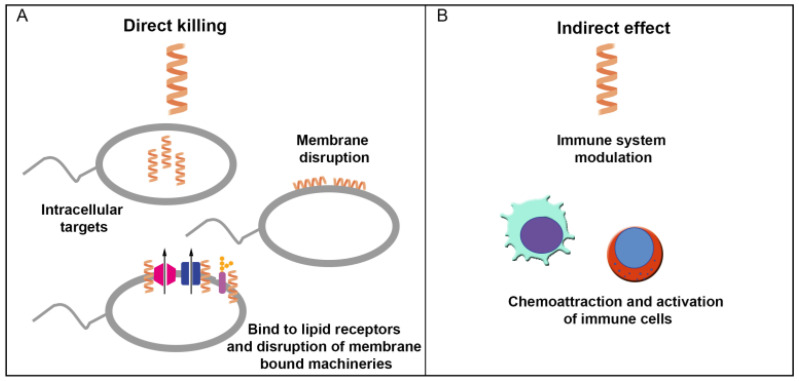
Antimicrobial peptide (AMP) mechanisms on bacterial cells: (**A**) AMPs directly affect bacterial membrane and intracellular targets and disrupt lipid receptors and membrane-bound machinery. (**B**) AMPs indirectly trigger the activation and chemoattraction of immune cells.

AMP activities begin on cell membranes through electrostatic interactions. As polycationic peptides, their multiple positive amino acids drive electrostatic interactions with lipid membranes that are also influenced by hydrophobic interactions (Figure 2, Table 2). Due to inherent differences between bacterial and mammalian cell surfaces, there would be preferences when AMPs associate with a cell surface, leading to an accumulation at the surface and self-assembly reaching a particular concentration [52,53]. At this stage, several models have been proposed to describe the mechanism of action (MOA) of AMPs.

**Figure 3:**
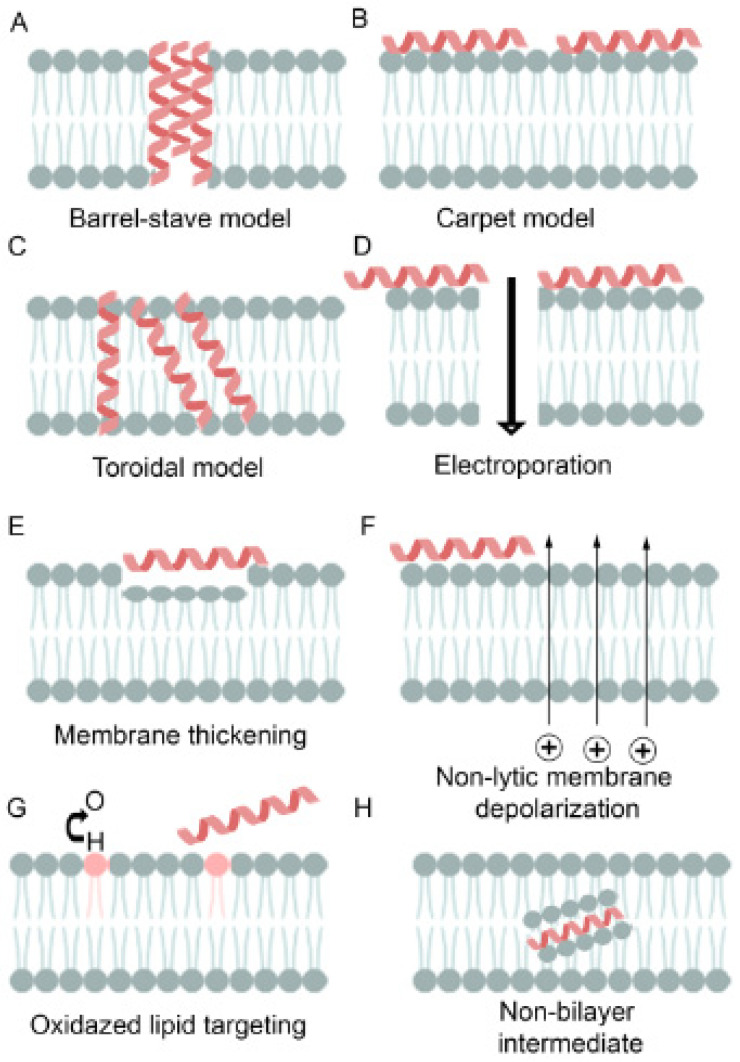
AMP mechanisms of action on bacterial membranes: (**A**) In the barrel-staved model, the accumulation of AMPs inserted into the membrane bilayer forms a pore. (**B**) In the carpet model, AMPs accumulate on the surface until a critical concentration displays detergent behavior to form micelles. (**C**) Accumulated AMPs inserted in vertical and bent orientations form a pore in the toroidal pore model. (**D**) Positively charged AMPs interact with negatively charged cell membranes adsorbing, leading to electroporation. (**E**) AMP interaction can interfere with membrane thickening, making the membrane more fragile. (**F**) Non-lytic membrane depolarization. (**G**) AMP oxidizes membrane lipids, leading to reactive oxygen species and increased lysis and permeability. (**H**) AMP generation of the non-bilayer intermediate that interacts with the membrane.

Multiple modes of action have been proposed for AMPs during interactions with bacterial cell surfaces, commonly known as transmembrane pore and non-pore models (Figure 3). The pore model presents differentiated forms such as barrel-stave and toroidal, reflecting the bilayer’s net arrangement. The barrel-stave shape preserves bilayer organization and begins as AMPs are parallel to the surface before perpendicularly inserting into the lipid bilayer [63]. The amphipathic structure of α and/or β sheet peptides permits lateral peptide–peptide interactions between hydrophilic amino acids to form the lumen, as well as the hydrophobic regions’ interaction with bilayer lipids [64,65], such as organizing and resembling a protein ion channel (Figure 3A). A minimum length of 22 residues in an α-helical structure or eight residues in a β sheet is needed to span a lipid bilayer. Only a subset of known AMPs, such as alamethicin [66], pardaxin [67,68], and protegrins [63], have been shown to form barrel-stave channels.

Toroidal pores also result from the perpendicular insertion of AMPs into the lipid bilayer but do not display lateral peptide–peptide interactions [66]. Rather, peptides disrupt the hydrophobic/hydrophilic arrangement of the bilayer and induce a local curvature in the lipid bilayer (Figure 3B). Pores are formed from a dynamic interaction between the inserted peptides and phospholipid head groups, creating a transient lipid–peptide supramolecule. In toroidal pores, the disruption in the hydrophobic and hydrophilic arrangement of the bills is temporary. Upon disintegration, some peptides are translocated to the inner cytoplasmic leaflet, allowing cell entry to target intracellular components [69]. Several AMPs, such as magainin 2 [70], lacticin Q [70], aurein 2.2 [71], and melittin [66,70], have been shown to form toroidal pores. For aurein 2.2, lipid composition and thickness have been shown to influence pore formation [72,73]. In a 1:1 mixture of 1-palmitoyl-2-oleoyl-sn-glycerol-3-phospho-(1-rac-glycerol) with 1-palmitoyl-2-oleoyl-sn-glycero-3-phosphocholine, toroidal pores are formed. In a membrane model of 1:1 1,2-dimyristoyl-sn-glycero-3-phospho-(1-rac-glycerol) with 1,2-dimyristoyl-sn-glyce rol-3-phosphocholine, aurein 2.2 does not form discrete pores. Other features of toroidal pores include ion and size selectivity [74]. Both toroidal and barrel-stave pores ultimately lead to membrane depolarization and cell death.

The carpet model describes AMPs that do not insert into the lipid bilayer to form pores [70,74,75,76]; peptides adsorb to the cell surface (Figure 3C). Upon reaching a threshold concentration, membrane integrity is compromised by a detergent-like effect that leads to the formation of micelles (Figure 3D). As the results in the carpet model are not dependent on specific amino acid compositions, lengths, or interactions, they can describe the MOA of several AMPs at high concentrations due to their amphiphilic nature, such as cecropin [77], indolicidin [78], aurein 1.2 [76], and LL-37 [75]. It has been suggested that the carpet-like mechanism is a prerequisite step for the toroidal pore model [71]. Other models have been proposed, including interfacial activity, electroporation, and Shai–Huang–Matsazuki models [71]. However, in most cases, the studies used the results from model membrane systems. Only a few AMPs have been studied in whole bacterial cells to define their MOAs [79,80], suggesting that the results from model membranes describing potential MOAs may need to explain their actions against the full pathogen.

Many AMPs are currently being studied to describe their therapeutic efficacy against *A. baumannii* strains. We have curated the online antimicrobial peptide database, APD3, to list the many examples of AMPs under study (Table 3 and Table 4). These include both peptides produced by living organisms and novel peptides inspired by their activities.

### 2.1. Cathelicidins

Cathelicidins are a group of more than 30 cationic AMPs (CAMPs) identified from the immune system of several vertebrates. Their structure comprises two domains involved in antimicrobial activity [214,215]. Cathelicidins have shown good activity compared to broad-spectrum carbapenems (imipenem and meropenem), antibiotics of choice to treat MDR *A. baumannii* (MIC = 16–32 mg/L) [216].

#### 2.1.1. Humans

The human cathelicidin LL-37 has an α-helical structure and is produced as a component of the innate immune response. It exhibits broad-spectrum microbicidal activity against Gram-positive and Gram-negative bacteria, observed as plasma–membrane disruptions [217]. It also neutralizes lipopolysaccharide (LPS) and modulates the immune response through cellular activation, inflammation regulation, chemotaxis, and wound healing [139,218,219,220,221]. LL-37 and its fragments KS-30 and KR-12 showed activity against four susceptible MDR *A. baumannii* clinical isolates [137]. LL-37 inhibited those five isolates at concentrations between 16 and 32 μg/mL; meanwhile, the minimum inhibitory concentration (MIC) for KS-30 and KR-12 was 8–16 and 128–256 μg/mL, respectively. In biofilms, LL-37 and KS-30 fragments significantly inhibited and dispersed *A. baumannii* on abiotic surfaces at 32 and 64 μg/mL, respectively [137].

LL-37-based synthetic peptides showed potent microbicidal activity against ESKAPE pathogens (*P. aeruginosa*, *A. baumannii*, and *S. aureus*) without selecting for resistance. They could eliminate persistent cells and biofilms at micromolar concentrations [164]. SAAP-148 is an α-helical AMP that is able to inhibit *A. baumannii* MDR growth and prevent biofilm formation at a concentration of 6 μg/mL. An ex vivo human skin infection model and an in vivo murine skin infection model eliminated acute and biofilm-related infections at concentrations of ~5% [164]. Its antibiofilm activity improved when incorporated into nanoparticles of Poly(lactic-co-glycolic) (PLGA) that gradually increased over time, suggesting a sustained local release of the peptide based on the dose–effect in vitro profile [105].

P10, a synthetic derivative of LL-37, is cationic, showing stronger activity than LL-37 [155,222]. The de novo pepD2, also LL-37-based, was designed as a trigonal distribution of positive charges in its helical structure. It displayed an 8 µg/mL MIC against the *A. baumannii* ATCC-type strain. WLBU2 (also called PLG0206) is an engineered cationic amphipathic α-helix, derived from LL-37 peptide, that can be inserted into bacterial membranes, leading to cell death as well as potent antibacterial effects against the biofilms of MDR *A. baumannii* and *K. pneumoniae* [168]. MIC values for WLBU2 were reported to be 1.5–3.2 μM for an XDR *A. baumannii* [223], 7.484 μM for clinical isolates [92], and 7.943 μM for *K. pneumoniae*.

#### 2.1.2. Snake

A large number of cathelicidins have been identified from snakes. Cathelicidin-BF (Cath-BF) was isolated from the venous glands of a banded krait (*Bungatus fasciatus*) [224]. It is one of the best-known cathelicidins, presenting an α-helical structure. Two mechanisms are attributed to its antimicrobial activity: disrupting bacteria membranes and directly pointing intracellular targets [224]. It has been proven to be highly active against the drug-resistant clinical isolates of *A. baumannii* and can inhibit growth at 12.8 μg/mL [105]. ZY4, a disulfide bridge, stabilized the cyclic peptide derivative of Cath-BF and displayed activity against clinical MDR isolates of *A. baumannii*, with MIC values ranging between 4.6 and 9.4 μg/mL. ZY4 killed bacteria via membrane permeabilization and inhibited biofilm formation [169]. With a half-life of 1.8 h in vivo, ZY4 displayed good stability and a low tendency to induce resistance. NA-CATH has an N-terminal α-helical structure with an unstructured C-terminal [85]. Identified from the Chinese cobra (*Naja atra*) [225], it can inhibit the growth of drug-resistant strains of *A. baumannii* at 10 µg/mL [225]. Its antimicrobial MOA appears to occur through membrane deformation and the formation of transient pores [226]. OH-CATH was identified from the king cobra (*Ophiophagus hannah*) [122]. Its analog, DOH-CATH30, exhibits microbicidal activity against MDR *A. baumannii* (1.56 to 12.5 μg/mL MIC).

#### 2.1.3. Alligator

The antibacterial activity of American alligator (*Alligator mississippiensis*) serum can be attributed to the presence of CAMPs, and several have been identified [227]. AM-CATH36 inhibited the growth of both drug-resistant and susceptible *A. baumannii* at 2.5 µg/mL, while its two fragments, AM-CATH28 and AM-CATH21, inhibited at 10 µg/mL [85]. All three appear to permeabilize bacterial membranes. MDR clinical isolates seemed more susceptible to the fragments than the full-length peptide. The recently identified As-CATH8 displayed in vitro activity profiles similar to the last-resort vancomycin and polymyxin B antibiotics. In a murine abscess model of high-density bacterial infections, As-CATH8 showed good activity against *A. baumannii* (MIC = 0.6 µg/mL) and *S. aureus* [86].

#### 2.1.4. Wallaby

WAM-1 is a cathelicidin in marsupial milk that is isolated from the Tammar wallaby (*Macropus eugenii*) mammary gland [167,228]. It inhibited biofilm formation in clinical isolates and dispersed the 24-hour-old biofilms of tested isolates, including MDR strains [89]. In comparison to LL-37, WAM-1 shows several desirable properties. WAM-1 in vitro activity was 12 to 80 times more effective than LL-37 at eliminating the clinical isolates of *A. baumannii*, and its activity as a peptide is not reduced in the presence of total serum or high NaCl concentrations. Although its MOA is unknown, it does not lead to hemolysis and has the potential for in vivo applications [89].

#### 2.1.5. Hoofed Animals

Domesticated animals have yielded several cathelicidins. Bovine neutrophils cytoplasmic granules contain indolicidin, a short tryptophan-rich cationic peptide that displaces divalent cations on the surface of cell membranes, forms pores, and can inhibit DNA-processing enzymes [90,229,230,231,232]. Indolicidin showed potent anti-*A. baumannii* activity (4–32 μg/mL MIC) on susceptible clinical isolates and 16 μg/mL against colistin-resistant strains [106]. In an in vitro combination with antimicrobial agents, indolicidin MIC was tested against 12 MDR clinical isolates and was reported to be between 2 and 64 μg/mL [90]. Bactenecin is a cyclic, arginine-rich cationic AMP isolated from cows, sheep, and goats with a type I β-turn structure and a disulfide bond between cysteines at positions 3 and 11 [90,133]. Bactenecin can make cell membranes more permeable and inhibit RNA and protein synthesis: 16 and 64 µg/mL MIC against susceptible and colistin-resistant *A. baumannii*, respectively [79,106]. Other studies of cathelicidins include bovine BMAP-27, sheep SMAP29, and goat minibactenecins [152], which have been shown to inhibit the growth of clinical MDR *A. baumannii* [93].

### 2.2. Defensins

Animals, plants, and fungi produce an ancient class of AMPs called defensins that contain six to eight conserved cysteine residues. Their MOA includes binding cell membranes, forming pores, and, consequently, killing pathogens [233]. Defensins have been categorized into α, β, and θ-defensins subfamilies [234].

#### 2.2.1. Human α-Defensins

The CAMPs HNP-1 and HNP-2 are α-defensins that are produced in human neutrophils that differ in their N-terminal amino acid. They are components of human neutrophil peptides in polymorphonuclear neutrophil granules released via secretion upon microbes’ activation [235]. *A. baumannii* ATCC 19606 was affected by 50 μg/mL MIC, while a colistin-resistant strain appeared more susceptible (MIC = 3.25 μg/mL) [106]. HD5, another human defensin, had little effect on *A. baumannii* (MIC = 320 μg/mL). However, its derivative HD5d5 showed a lower MIC (40 μg/mL) through cell membrane damage and cell entry, reducing superoxide dismutase and catalase activities [132,236].

#### 2.2.2. β-Defensins

HBD-2 and HBD-3 are human β-defensins found on the epithelial lining of respiratory and urinary tracts. Interestingly, they appear more effective against MDR clinical isolates [237]. The other β-defensin, HBD-3, combines an α-helical segment with a β strand and can kill non-MDR and MDR *A. baumannii* isolates in serum-free conditions [238]. HBD-3 also showed wound-healing properties and a potential application in wound dressings [131,239]. In *A. mississippiensis*, the AM23sk isoform of HBD-3 β-Defensin showed in vitro antibacterial activity against *A. baumannii* (MIC = 2 μg/mL) [86].

#### 2.2.3. Insect Defensins

The insect defensin, CL-defensin, can partially permeabilize *A. baumannii* and, different from others, is predicted to have an N-terminal loop, an α-helix segment, and an antiparallel β-sheet according to circular dichroism spectroscopy [109].

### 2.3. Frog AMP

#### 2.3.1. Magainin and Pexiganan

The skin of the African clawed frog (*Xenopus laevis*) has two α-helical cationic amphipathic AMPs, Magainin-1 and Magainin-2 [240]. Their primary MOA against microbes is pore formation [101,241]. Magainin-2 shows higher activity against MDR *A. baumannii* (4.9–64 μg/mL) and can inhibit isolates and eliminate biofilms [101,106]. It also offers greater stability in physiological conditions and low hemolytic activity. Magainin-2 shows anticancer potential and low toxicity against non-cancerous mammalian cells [85]. A synthetic analog of Magainin-2, Pexiganan, or MSI-78 also displays a broad potent action against the formation of toroidal pores [242,243,244]. Pexiganan can inhibit the growth of clinical MDR *A. baumannii* at 1–8 μg/mL [159,160,245]. Studies testing ATCC 196060, a reference strain of *A. baumannii*, confirmed pexiganan’s antimicrobial and antibiofilm activity [88].

#### 2.3.2. Brevinin-2 Related Peptide

Skin secretions from both mink frog (*Rana septentrionalis*) and carpenter frog (*R. virgatipes*) contain B2RP, a brevinin-related AMP with an α-helical structure that affects bacterial membrane organization [246,247]. B2RP can inhibit susceptible *A. baumannii* (29 μg/mL) and MDR isolates (7–13.9 μg/mL) [98]. However, its hemolytic properties limit its potential use [248]. Three analogs of B2RP (D4K, K16A, and L18K) showed reduced red blood cell toxicity and a two-fold increase in activity against *A. baumannii* growth [96,98]. The D4K substitution also showed activity against colistin-resistant and XDR *A. baumannii* clinical isolates [120]. B2RP-ERa is a smaller cationic peptide that is structurally similar to B2RP found in the skin of Asian frogs (*Hylarana erythraea*) [97,249]. It can inhibit susceptible *Acinetobacter* growth 8–32 μg/mL and drug resistant (8–64 μg/mL) [96]. B2RP-ERa shows anti-inflammatory characteristics without toxicity on peripheral blood mononuclear cells or red blood cells [249,250].

#### 2.3.3. Alyteserins

Alyteserin-1c and Alyteserin-2a are two cationic AMPs that show that anti-*A. baumannii* activity is released from the skin secretions of midwife toads (*Alytes obstetricans*) following norepinephrine stimulation [83,165,251]. Alyteserin-1c inhibited MDR *A. baumannii* growth and caused death between 11.3 and 22.6 μg/mL, showing low hemolytic activity [83]. The substitution E4K further reduced the effects on red blood cells while improving growth inhibition of colistin-resistant and XDR *A. baumannii* isolates [120]. Structural changes of Alyteserin-2a also resulted in an analog with 4–8-fold greater antimicrobial activity and less hemolytic effects [95].

#### 2.3.4. Peptide Glycine–Leucine-Amide

The volcano-clawed frog (*Xenopus amieti*) produces PGLa-AM1, peptide glycine–leucine-amide, an AMP with anti-*Acinetobacter* activity. PGLa-AM1 can kill susceptible and colistin-resistant *A. baumannii* (16–128 μg/mL) [161]. Due to its low hemolytic activity, it is also active against other ESKAPE pathogens, including *E. coli* and *S. aureus* [81,111,161].

#### 2.3.5. Caerulein Precursor Fragment

Also isolated from the volcano-clawed frog, the caerulein precursor fragment (CPF-AM1) is a cationic AMP that binds bacterial LPS [97,120]. CPF-AM1 inhibits the growth of susceptible and colistin-resistant isolates, showing minimal fibroblast toxicity and hemolytic activity [136]. CPF-B1 was isolated from a Marsabit clawed frog (*Xenopus borealis*), displaying anti-*A. baumannii* activity at concentrations between 11.4 and 22.8 μg/mL and low hemolysis [113]. From the Peracca clawed frog (*Xenopus clivii*), CPF-C1 is another member of the caerulein family of peptides with proven activity against *A. baumannii*, including inhibitory activity as low as 5 μg/mL concentration [112].

#### 2.3.6. Hymenochirins

Hymenochirin-1B was isolated from a Zaire Dwarf clawed frog (*Hymenochirus boettgeri*) and is the first member of the hymenochirins class of AMPs of their host defense system [252,253]. Hymenochirin-1B is an α-helical cationic peptide able to inhibit the growth of MDR *A. baumannii* at 19.1 μg/mL MIC [123]. In addition to its antimicrobial activity, it displays anticancer and immunomodulatory properties. Hymenochirin-1B, generated by E6K and D9K substitutions, showed a nearly 4-fold increase in activity against MDR and XDR *A. baumannii* isolates and reduced toxicity to human erythrocytes [123]. Hymenochirin-1Pa was isolated from Merlin’s dwarf gray frog (*Pseudhymenochirus merlini*) and was able to inhibit the growth of XDR *A. baumannii* between 7.5 and 15 μg/mL; however, it showed moderate hemolytic activity [136,253].

#### 2.3.7. XT-7

Skin norepinephrine stimulation allows secretion of XT-7 from Western clawed frog (*Xenopus tropicalis*) [254], an AMP with anti-*Acinetobacter* activity against the Euroclone I NM8 strain at 22.2 μg/mL MIC [112]. A G4K substitution increased XT-7 therapeutical index [128], inhibiting susceptible and drug-resistant *A. baumannii* by as low as 4 μg/mL [161].

#### 2.3.8. Buforins

The stomach of an Asian toad (*Bufo gargarizans*) yielded Burfoin I [255]. Its derivative, Buforin II, is a potent antimicrobial peptide that kills bacteria by crossing the membrane to bind intracellular targets, including DNA and RNA, inhibiting cellular activities [102]. Buforin II can hinder the growth of susceptible and resistant *Acinetobacter* isolates between 0.25 and 39 μg/mL [100,101]. By itself, or in combination with antibiotic treatments, Buforin II demonstrated good potential when tested in an *A. baumannii* rat sepsis model [96].

#### 2.3.9. Caerin 1.1 and 1.9

The host defense peptides caerin 1.1 and caerin 1.9 from an Australian tree frog (*Litoria caerulea*) were isolated from skin secretion. They are α-helical cationic amphipathic AMPs with antiviral, antitumor, antimicrobial, and neuropeptide-type activities [256]. Each displayed anti-*A. baumannii* growth activity was more effective when in combination [104].

#### 2.3.10. Hylin a1

Hylin a1 is an α-helical cationic amphipathic AMP that was isolated from the skin secretion of a white spotted tree frog (*Hypsiboas albopunctatus*) [257]. Its antimicrobial activity has been attributed to its action on bacterial membranes. However, it also displays a strong hemolytic activity. Two analogs, Hylin a1-11K and Hylin a1-15K, showed good antimicrobial activity against carbapenem-resistant *A. baumannii* clinical isolates at 1–2 µM without changes in hemolytic activity [82].

### 2.4. Fish Piscins

Fish possess a strong innate immune system as a first-line defense against various pathogens [258]. Several antimicrobial components can be found within the epidermal mucus, including AMPs, lysozyme, proteases, and lectins [259]. Piscidins are cationic AMPs expressed by fish mast cells [260], which comprise a family of structurally related mature peptides between 21 and 44 residues. They are made of an amphipathic α-helical structure, which suggests that piscins have bactericidal activities against microorganisms [261]. The piscidin AMP family includes pleurocidin, moronecidin, chrysophsin, dicentracin, epinecidin-1, and myxinidin [262].

Pleurocidin is an amphipathic α-helical cationic peptide found in the gills, gut, and on the skin of winter flounder (*Pseudopleuronectes americanus*) [263], which is genetically similar to piscidin [264]. It displays broad-spectrum activity against pathogenic bacteria and fungi such as *K. pneumoniae*, *S. aureus*, *P. aeruginosa*, and the opportunistic oral pathogen *C. albicans* [263,265]. Against MDR *A. baumannii* isolates, pleurocidin inhibits growth between 8 and 32 μg/mL [93]. Its MOA appears to be caused by membrane disruption due to its binding [266]; however, it shows lower hemolysis when compared to other natural peptides using in vitro toxicity studies [267].

Tilapia piscidin 2 (TP2) is an inactive antibacterial peptide found in Nile tilapia (*Oreochromis niloticus*) [268], which was modified to develop peptides TP2-5 and TP2-6, improving cationic and amphipathic balance [269]. Such changes resulted in a significant improvement in their antimicrobial potential in normal media against *A. baumannii* wild-type (MIC = 3.1 μg/mL) and MDR isolates (MIC = 1.6–12.5 μg/mL) [211]. Another AMP from Nile tilapia (TP4) displayed antimicrobial activity against susceptible and MDR *A. baumannii* between 16 and 32 μg/mL MIC [97].

### 2.5. Hepcidin

First identified from human ultrafiltrate blood and urine samples and called a liver-expressed antimicrobial peptide (LEAP-1) [270,271], hepcidin is a cationic amphipathic peptide that functions in many vertebrates. Hepcidin reportedly involves iron metabolism, inflammation, and clearance of invading pathogens [272]. Since the first fish hepcidin was reported in hybrid striped bass in 2002 [273], many isoforms have been identified across numerous fish species. Unlike a single gene in humans, many teleost fish have more than two hepcidin genes, most notably among Perciformes and Pleuronectiformes [274]. Fish hepcidin isoforms are currently phylogenetically classified into HAMP1-type and HAMP2-type [275,276,277]. From Japanese seabass (*Lateolabrax japonicus*), LJ-hep2 peptide has been investigated using its recombinant precursor protein (rLJ-hep2), which is expressed in *Pichia pastoris* and is a chemically synthesized mature peptide LJ-hep2 (66–86), with LJ-hep2 (66–86) displaying stronger antimicrobial activity against clinically isolated MDR *A. baumannii* (MIC = 1.5–3 μg/mL) [179].

### 2.6. Melittin

The cationic amphipathic α-helical AMP melittin was isolated from European honeybee (*Apis mellifera*) venom, comprising nearly half its dry weight [278]. Numerous melittin properties have been reported, including antibacterial [278], antiparasitic [279], and antifungal [280], along with anticancer and antiviral properties [281]. Its primary MOA is a carpet-like interaction with membranes, leading to pore formation and lysis [282]. Melittin displays potent antimicrobial activity against clinical MDR and XDR *Acinetobacter* at concentrations as low as 0.125 μg/mL [149,150]. In a mouse model of third-degree burns, the topical application of melittin at 16 µg/mL eliminated 93.3% of an XDR *A. baumannii* [149]. Importantly, the injured derma and surrounding tissue, including red blood cells, showed no toxicity. Brazilian clinical studies confirmed melittin activity against most *Acinetobacter* strains except for one PDR [283].

Trypsin Modulating Oostatic Factor (AeaTMOF) is a proline-rich amphipathic decapeptide that is analogous to PrAMP, which was first reported in honeybees [284]. AeaTMOF (5 mM) was very effective against *A. baumannii,* inhibiting cell growth during 15 h incubation [285].

### 2.7. Cecropins

Cecropin describes a class of AMPs in which primary MOA is attributed to membrane lysis [286]. The founding compound, cecropin A, was isolated from giant silk moth (*Hyalophora cecropia*) hemolymph [287]. Initial results showed in vitro antibacterial and anticancer activity [288]. Viability studies performed in the *Caenorhabditis elegans* model on *A. baumannii* infections demonstrated that 15 cecropin or cecropin-like peptides displayed antimicrobial activity and improved survival [99]. Several other studies have further defined the growth inhibition of individual peptides, including cecropin A against colistin-resistant MDR clinical isolates [106,107], BR003-cecropin A (from *Aedes aegypti*) against MDR *A. baumannii* [99], Musca domestica cecropin (Mdc) from housefly (*Musca domestica*) larvae against standard and MDR isolates [148], cecropin-4 from houseflies against MDR and XDR clinical isolates [172,173,205], and cecropin P1 from pig roundworms (*Ascaris suum*) against colistin-susceptible *A. baumannii* [106]. Many cecropins also display antibiofilm activity, such as myxinidin isolated from hagfish (*Myxine glutinosa*) [153] and the AMP complex Fly Larvae Immune Peptides 7 (FLIP7) in blowfly (*Calliphora vicina*) larvae [127].

The fusion of cecropin A to endolysin ST01 has been shown to have increased bactericidal activity against ESKAPE pathogens, with *A. baumannii* (ATCC 17978) being eliminated at a concentration of 0.25 [289]. Another hybrid of cecropin with melittin, CAMEL, rapidly kills *A. baumannii* [88]. OMN6 is a 40-amino acid synthetic cyclic peptide based on cecropin A that displays increased stability and a significant decrease in proteolytic degradation and low cytotoxicity against eukaryotic cells. This peptide exerts a rapid bactericidal effect causing a selective bacterial membrane disruption [195], which is effective in *A. baumannii* laboratory (MIC = 8 μg/mL) and clinical isolates (MIC = 4–8 μg/mL), suggesting a low likelihood for resistance development [195].

### 2.8. Mastoparan

Mastoparan was isolated from hornet (*Vespula lewisii*) venom [236]. While it displays good activity against wild-type, colistin-resistant, and PDR clinical *A. baumannii* [106,290], it also shows high hemolytic activity, which would prevent its therapeutic application [291]. Action against clinical MDR *A. baumannii* (2–16 μg/mL MIC) was observed for mastoparan-AF isolated from *Vespa affinis* [148]. Improvements in serum stability (24 h) have been achieved for mastoparan analogs, resulting in the growth inhibition of XDR clinical isolates [106]. Higher therapeutic efficiency against MDR clinical isolates has been acquired by conjugating mastoparan to chitin, resulting in nanoconstructs (*Afreenish hassan*). Improvements in hemolytic toxicity have not been reported.

### 2.9. Histatins

Histatins are a family of low-molecular-weight, histidine-rich cationic peptides isolated from salivary glands, which display antimicrobial activity through membrane disruption [240]. The only member that affects *A. baumannii* is histatin-8, a hemagglutination-inhibiting peptide [248]. It inhibited the growth of colistin-susceptible and colistin-resistant isolates at 32 μg/mL [106].

### 2.10. Dermcidin

The *dcd* gene in humans encodes dermcidin, a two-region anionic AMP produced and secreted by eccrine sweat glands and transported to the skin surface [116,292]. The N-terminal peptide is involved with neuronal cell survival in response to oxidative stress [116]; meanwhile, the C-terminal fragment shows anti-*Acinetobacter* activity [293]. With a net charge of -2, DCD-1L can interact with negatively charged bacterial phospholipids. Clinical PDR *A. baumannii* shows a two-fold increase in susceptibility compared to XDR isolates and the standard ATCC 19606 strain [117]. DCD-1L can also inhibit bacterial attachment and biofilm formation, which could affect infection initiation [117].

### 2.11. Tachyplesin III

The hemolymph of Southeast Asian horseshoe crabs (*Tachypleu gigas* and *Carcinoscorpius rotundicauda*) contains tachyplesin III and 17 amino acids AMP. As opposed to an α-helical structure, this peptide presents a cyclic β-sheet with two disulfide bridges. Against an XDR clinical *A. baumannii*, tachyplesin III had 8–16 μg/mL MIC and could fully eliminate the bacteria at twice the MIC concentration [294]. However, it also displays high toxicity against mammalian cells, preventing therapeutic applications [294].

### 2.12. Spider Peptides

Several AMPs have been isolated from spider venom. Lycosin-I is a 23-amino acid peptide from a Chinese wolf spider (*Lycosa singoriensis*) venom, resulting in 8–32 µg/mL MIC against MDR *A. baumannii* [140,295]. Ant spider venom (*Lachesana tarabaevi*) and latarcins 2a also displayed potent antimicrobial activity against clinical MDR *A. baumannii* (8–64 μg/mL) [93]. Like Lycosin-I, LS-AMP-E1, and LS-AMP-F1, those from burrowing wolf spiders (*Lycosa sinensis*) had different inhibitory activity against other clinical drug-resistant bacteria and could effectively inhibit the formation of biofilms with no obvious hemolytic effects. Among ESKAPE pathogens, LS-AMP-F1 was the most effective against *A. baumannii*, with the lowest being 3.1 µM MIC [140]. LyeTx I was isolated from a wolf spider from Brazil (*Lycosa erythrognatha*) and showed inhibitory activity against several MDR bacteria. However, it also showed hemolytic and cytotoxic effects. Conjugating a derivative, LyeTx I-b, to PEG could eliminate these contradictory effects while maintaining MIC values against *A. baumannii*, such as antibiofilm formation, and did not induce resistance [186].

### 2.13. Scorpion

Many AMPs have been identified from scorpion venom, displaying antimicrobial activity against *A. baumannii*, such as Hp1404, ctriporin, and Im5 [94]. Notably, these peptides also show harmful effects, such as hemolysis, requiring sequence alterations to fix. Hp1404 was isolated from the venomous gland of a giant forest scorpion (*Heterometrus petersii*) and is an amphipathic α-helical peptide that exhibits antimicrobial activity against methicillin-resistant *S. aureus* along with cytotoxicity. Many Hp1404 analogs showed lower cytotoxic activity against MDR *A. baumannii* [134]. BmKn2 is another naturally occurring cationic α-helical AMP in the Chinese scorpion (*Mesobuthus martensii Karsch*), showing antimicrobial and strong hemolytic activity. It only shows activity against Gram-positive bacteria, such as *S. aureus*. Its mutant BmKn2-7 has lower hemolytic activity and presents a broad antimicrobial spectrum [296]. Another analog, BmKn2-7K, is non-toxic at antimicrobial dosages and exhibits potent antimicrobial activity via a membrane-lytic mechanism against antibiotic-resistant ESKAPE pathogens. For MDR *A. baumannii*, BmKn2-7K and BmKn2-7R (MIC = 2.5–5 µg/mL) showed potent and improved antimicrobial activity compared to BmKn2-7 (MIC = 5–10 µg/mL) [95].

### 2.14. Lynronne-1

Lynronne-1 is an α-helical cationic amphipathic peptide identified through the metagenomic investigation of bovine rumen microbiome to discover novel AMPs. Although Lynronne-1 in vivo activity was lower than conventional antibiotics, it showed selectivity for bacterial cells, low hemolytic activity, and minimal cytotoxicity against mammalian cells [297]. Against most common Gram-positive and Gram-negative pathogenic bacteria, Lynronne-1 displayed broad-spectrum activity, including methicillin-resistant *S. aureus* (MRSA) 8–32 μg/mL MIC and *A. baumannii* (4 μg/mL MIC) [297].

### 2.15. Hybrid Peptides

The combination of different AMPs offers a rational approach to developing non-natural AMPs. PapMA peptide consists of 18 amino acids, combining the first eight amino acids from papiliocin, a 37-residue AMP purified from the larvae of a swallowtail butterfly (*Papilio xuthus*) with resides 4–12 of magainin 2, and a 23-residue AMP purified from African clawed frog (*Xenopus laevis*) skin. A proline hinge joined the two fragments. While PapMA showed high antimicrobial activity, it was cytotoxic to mammalian cells [298]. The hybrid peptides P7A3 and A3P7 that combined cathelicidin (P7) and aurein (A3) were obtained using the flipping technique [299]. The serial truncation of the C-terminal led to an optimal candidate, AP19, that was stable against proteolytic enzymes via a D-amino acid substitution (D-AP19). The final peptide rapidly killed *A. baumannii* ATCC 19606 (MIC = 7.81 µg/mL) via membrane disruption and showed a low tendency to induce bacterial resistance. It also exhibited potent antibacterial activity against MDR and XDR *A. baumannii* (MIC = 3.91–15.63) [176]. BP214 is a cationic amphipathic all-D decapeptide developed from a short cecropin A-melittin hybrid peptide BP100 [300], which showed excellent activity against colistin-resistant *A. baumannii* and modest hemolytic properties [301].

## 3. Resistance to AMPS

Resistance to AMPs can be acquired through their degradation, sequestration, and impedance by exopolymers or biofilm matrix molecules, as well as through the alteration of membrane lipid composition and exporting mechanisms [53,302,303,304,305,306,307] (Table 5). Following its long-term clinical use, colistin resistance has been documented for *A. baumannii* [308,309]. Resistance was also observed after inactivating one of the genes involved in LPS biosynthesis. As colistin is a last-resource drug to treat MDR nosocomial pathogens, resistance is an important clinical issue [309,310,311]. Several nanocarriers have been developed to overcome low bioavailability, proteolysis, and toxicity associated with AMPs [312,313]. Changes in molecular structure, biochemical modifications, and their combination with common antibiotics have been reported to minimize AMP resistance [303].

## 4. Conclusions

Among ESKAPE pathogens, *A. baumannii* is of major concern for nosocomial and community-acquired infections. Due to its high ability to acquire resistance and biofilm formation, there has been an alarming loss of antibiotic efficacy and a rise in MDR isolates worldwide. The shortage of new antibiotic treatments shows the need to transition to a “post-antibiotic era” by developing new alternative therapeutical approaches. AMPs have emerged as excellent candidates due to the broadness of natural peptides found as part of innate immune systems, demonstrating activity against many *A. baumannii*, including clinical MDR and XDR isolates. While many AMPs display undesirable effects, such as hemolysis and host toxicity, studies have demonstrated the ability to modify their sequences to improve performance. AMPs isolated from natural sources have attracted significant interest in recent years as promising pharmacological substitutes for conventional antibiotics; moreover, extensive research has been undertaken on the discovery, production, and optimization of peptide drugs. Future advances in bioinformatics and studies on peptide sequence/structure/function could be able to develop synthetic AMPs to address major health concerns. Our review of AMPs highlighted common characteristics, such as cationic, α-helical structure, interactions with bacterial membranes, bilipid pore formation, and intracellular component targeting. Many possibilities for performance improvement combined with traditional treatments and their use as bioconjugates encourage future applications. Peptide drugs currently represent a significant proportion of the pharmaceutical market. Considering their therapeutic potential, market prospects, and economic values, antimicrobial peptides are expected to attract investments and research efforts, achieving success in the medium to long term. In addition to their antimicrobial properties, many AMPs have demonstrated other beneficial activities such as anticancer, antioxidant, wound healing, and angiogenesis that further support additional research.

## Figures and Tables

**Table 2:** General mechanism of AMP actions: direct killing by inhibiting membranes, bacterial lysis, and immune modulation.

Mechanism of AMP	Mode of Action	Reference
Direct killing: Membrane target	Electrostatic interactions and hydrophobic interactions (peptide and bacterial cell surface), membrane rupture-bound types of machinery and bacterial lysis—bilayer disruption	[54,55,56,57,58,59]
Direct killing: Non-membrane target	Action on the bacterial cell wall, activation of autolysin, intracellular targets: inhibition of protein/nucleic acid synthesis, disruption of enzymatic activities and bacterial lysis	[54,55,56,57,60]
Immune modulation	Chemotaxis, activation of immunocytes, microbial killing; anti-endotoxin activity, suppression of toll-like receptors (TLRs) and/or cytokine-mediated production of proinflammatory cytokines and preventing excessive and harmful proinflammatory responses, controls the inflammation	[57,61,62]

**Table 3:** AMPs produced by living organisms demonstrating anti-*A. baumannii* activity.

Peptide	Source	Sequence (nº Amino Acid)	Structure	MIC against *A. baumannii* (μg/mL)		Ref.
				ATBS	MDR	
Agelaia-MPI	*Agelaia pallipes pallipes*	INWLKLGKAIIDAL (14aa)	AH	6.25	12.5–25	[81]
aHylin a1-15K	*Hypsiboas albopunctatus* (American frog)	IAKAILPLALKALKKLIK-NH2 (19aa)	AH	1–2 *	1–2 *	[82]
Alytesirin-1c	Frog skin peptide	GLKEIFKAGLGSLVKGIAAHVAS-NH2 (23aa)	AH	—	11.3–22.6	[83]
α-Helical-26 (A12L/A20L)	D- and L-diastereomeric peptides	Ac KWKSFLKTFKSLKKTVLHTL LKAISS-NH2 (26aa)	AH	—	0.5–1.0	[84]
AM-CATH21		GLFKKLRRKIKKGFKKIFKRL (21aa)	AH	42	10	[85]
AM-CATH28	American alligator	KIKKGFKKIFKRLPPIGVGVSIPLAGKR (28aa)	AH	28	10	
AM-CATH36		GLFKKLRRKIKKGFKKIFKRLPPIG VGVSIPLAGKR (36aa)	AH	5.2	5.2	
Am23SK	Alligator mississippiensis	SCRFSGGYCIWNWERCRSGHFLVALCPFRKRCCK (34aa)	AH	—	2	[86]
Artlysin Art-175	*Pseudomonas aeruginosa* bacteriophage	Comprises a modified variant of endolysin KZ144 with an N-terminal fusion to SMAP-29	NF	—	4–20	[87]
Aurein 1,2	Frog skin peptide	GLFDIIKKIAESF (13aa)	AH	16	—	[88]
Bactenecin	Bovine neutrophil granules, Caprine	LCRIVVIRVCR (12aa)	B-turn structure Ciclyc	64	—	[89,90,91]
Bicarinalin (YRTX-Tb1a)	Tetramorium bicarinatum venom	KIKIPWGKVKDFLVGGMKAV (20aa)	AH	—	4	[92]
BMAP-27	Bovine myeloid	GRFKRFRKKFKKLFKKLSPVIPLLHLG (27aa)	AH	8–16	4–16	[93]
BmKn1	*Heterometrus petersii* (Scorpion venom gland)	FIGAVAGLLSKIF (13aa)	AH	>40	—	[94]
BmKn2	*Mesobuthus martensii Karsch* (Scorpion)	FIGAIARLLSKIF-NH2 (13aa)	AH	10	5–10	[95]
B2RP-Era	Frog skin peptide	GVIKSVLKGVAKTVALGML-NH2 (19aa)	AH	8–32	8–64	[96,97]
Brevinina 2 (B2RP)	Frog skin peptide	GIWDTIKSMGKVFAGKILQNL-NH2 (21aa)	AH	29	7–13.9	[98]
BR003-cecropin A	*Aedes aegypti*	GGLKKLGKKLEGAGKRVFNAAEK ALPVVAGAKALRK (36aa)	AH	5	5	[99]
Buforin II	Frog skin peptide	TRSSRAGLQFPVGRVHRLLRK (21aa)	AH	8–19.5	0.25–39	[100,101,102,103]
Caerin 1.1		GLLSVLGSVAKHVLPHVVPVIAEHL-NH2 (25aa)	AH	7.5	—	[104]
Caerin 1.9	Australian tree frog	GLFGVLGSIAKHVLPHVVPVIAEKL-NH2		3.75	—	
Caerin 1.1 + Caerin 1.9		GLLSVLGSVAKHVLPHVVPVIAEHL-NH2+ GLFGVLGSIAKHVLPHVVPVIAEKL-NH2		0.9375–1.875	—	
CATH-BF derivative (Cath-A and OH-	*Bungarus fasciatus* (Snake venom)	KFFRKLKKSVKKRAKEFFKKPRVI GVSIPF(30aa)	AH	—	8–32	[105]
Cecropin A	*Hyalophora cecropia* (Cecropia moth)	KWKLFKKIEKVGQNIRDGIIKAGP AVAVVGQATQIAK (37aa)	AH	32	0.5–32	[106,107]
Cecropin P1	*Ascaris suum* (Pig)	SWLSKTAKKLENSAKKRISEGIAIA IQGGPR (31aa)	AH	1.6	—	[106,108]
Citropin 1.1.	*Litora genus* (Australian tree frog)	GLFDVIKKVASVIGGL-NH2 (16aa)	AH	16	—	[88]
CL defensin	*Cimex Lectularius* (Bedbug)	ATCDLFSFQSKWVTPNHAACAAHCTARGNRGGRCKKAVCHCRK (43aa)	AH, antiparallel BS; N-terminal loop	—	—	[109]
Colistin (Polymyxin E)	*Bacillus colistinus*	C52H98N16O13 (cyclic compound)	*BS* n and B-tur	Antibiofilm, side effects	—	[110]
Con10	*Opisthacanthus cayaporum* (Scorpion venoms)	FWSFLVKAASKILPSLIGGGDDNKSSS (27aa)	AH	12.5	12.5	[81]
CPF-AM1	Frog skin peptide	GLGSVLGKALKIGANLL (19aa)	AH	16–128	4–128	[96,111,112]
CPF-B1	Frog skin peptide	GLGSLLGKAFKIGLKTVGKMMGGAPREQ (28aa)	AH	—	11.4–22.8	[113]
CPF-C1	Frog skin peptide	GFGSLLGKALRLGANVL (17aa)	AH	5	—	[112]
Ctriporin	*Heterometrus petersii* (Scorpion venom gland)	FLWGLIPGAISAVTSLIKK (19aa)	AH	20	20–40	[94]
Cy02 (cyclotide)	Viola odorata	GIPCGESCVWIPCISSAIGCSCKSKVCYRN (30aa)	BSs	—	15 *	[114]
Danalexin	*American bulfrog* (Rana catesbeiana)	LGGLIKIVPAMICAVTKKC (19aa)	AH	—	4–16	[115]
DCD-1 L	Eccrine sweat glands	SSLLEKGLDGAKKAVGGLGKLGKDAVEDLESVGKGAVHDVKDVLD SVL (48aa)	AH	16	—	[116,117]
D-150-177C, HBcARD derivative peptide	Hepatitis B virus	RRRGRSPRRRTPSPRRRRSQSPRR RRSC (28aa)	AH	16	16–32	[118]
Delfibactin A	Gram-negative bactéria *Delfia* spp.	C40H68N14O18	NF	—	16	[119]
[D4K] B2RP	Frog skin peptide	GIWKTIKSMGKVFAGKILQNL-NH2 (21aa)	AH	4–16	4–16	[96,120]
D-Myrtoxin-Mp 1a (Mp1a)	*Myrmecia pilosula* (Venom)	IDWKKVDWKKVSKKTCKVMXKACKEL-NH2 (26aa)	AH	0.025 *	—	[121]
DOH-CATH30	King cobra (Snake venon)	KFFKKLKNSVKKRAKKFFKKPRVIGVSIPF (30aa)	AH	—	1.56–12.5	[122]
[E4k] Alytesirin-1c	Frog skin peptide	GLKEIFKAGLGSLVKGIAAHVAS-NH2 (23aa)	AH	4–16	4–16	[96,120]
[E6k,D9k] Hymenochirin-1B	Frog skin peptide	LKLSPKTKDTLKKVLKGAIKGAIA IASMA-NH2 (29aa)	AH	—	4.9	[123]
Epi-122–42	*Epinephelus coioides* (Orange-spotted grouper)	GFIFHIIKGLFHAGKMIHGLV (21aa)	NF	—	4–32	[124]
Epsilon-poly L-lysine (EPL)-catechol	*Streptomyces albulus* derived	Complex	NF	—	Reducing bacterial burden in vivo	[125]
Esc(1-21)	Frog-skin	GIFSKLAGKKIKNLLISGLKG-NH2 (21aa)	AH	—	17.5–35	[126]
Esc(1-21) + Colistina		GIFSKLAGKKIKNLLISGLKG-NH2 (21aa) + Colistina	AH	—	1.1–4.4	
FLIP 7	*Calliphora vicina* (Medicinal Maggots)	ATCDLLSGTGANHSACAAHCLLRGNRGGYCNGKAVCVCRN (40aa)	AH	—	125–416 biofilm bactéria sensitivity	[127]
[G4K] XT7	Frog skin peptide	GLLGPLLKIAAKVGSNLL-NH2 (18aa)	AH	4–32	4–64	[96,128]
Glatiramer acetate	*Homo sapiens*	EAYKAAEKAYAAKEAAKEAAKAKAEKKAAYAKAKAAKYEKKAKKAAAEYKKK (52aa)	NF	Reduct viable cells	Reduct viable cells	[129]
HBD-2	The epithelial lining of the respiratory/urinary tracts	GIGDPVTCLKSGAICHPVFCPRRYKQIGTCGLPGTKCCKKP (41aa)	Beta	3.90–9.35	3.25–4.5	[130]
HBD-3	The epithelial lining of the respiratory/urinary tracts	GIINTLQKYYCRVRGGRCAVLSCLPKEEQIGKCSTRGRKCCRRKK (45aa)	AH + BS	4	4	[131]
HBD-3	Epithelial cells	GIINTLQKYYCRVRGGRCAVLSCLPKEEQIGKCSTRGRKCCRRKK (45aa)	AH + BS	—	4–16	[124]
HD5d5	*Homo sapiens* (Polymorphonuclear neutrophil)	ARARCRRGRAARRRRLRGVCRIRGRLRRLAAR (32aa)	AH	40	40	[132]
Histatin-8	*Homo* sapiens	KFHEKHHSHRGY (12aa)	AH	8	—	[133]
HNP-1	*Homo sapiens* (Polymorphonuclear	ACYCRIPACIAGERRYGTCIYQGRLWAFCC (30aa)	AH	50	—	[106]
HNP-2	(neutrophil)	CYCRIPACIAGERRYGTCIYQGRLWAFCC (29aa)		50	—	[106]
Hp1404	*Heterometrus petersii* (Scorpion venom gland)	GILGKLWEGVKSIF (14aa)	AH	5	5–10	[94,134]
Hp1404 analogs		GILGKLWEGVKSIF (14aa) analogs		3.13–25 *	—	
Hp l404 analogs (A, K, V, L, I, W)	*Heterometrus petersii* (Scorpion venom gland)	GILGKLWEGVKSIF-NH2 (14aa)	AH	3.13–12.5	3.13–16.25	[134,135]
Hylin a1	*Hypsiboas albopunctatus* (American frog)	IFGAILPLALGALKNLIK-NH2 (18aa)	AH	2 *	2–8 *	[82]
Hylin a1-11K		IAKAILPLALKALKNLIK-NH2 (19aa)		1–2*	1–2 *	
Hymenochirin-1 Pa	Frog skin peptide	LKLSPKTKDTLKKVLKGAIKGAIAIASMA-NH2 (29aa)	AH	—	—	[136]
Im4	*Heterometrus petersii* (Scorpion venom gland)	FIGMIPGLIGGLISAIK (17aa)	AH	>40	—	[94]
Im5		FLGSLFSIGSKLLPGVIKLFQRKKQ (25aa)	AH	2.5	2.5–10	
Indolicidin	Cytoplasmic granules of the bovine neutrophils	LPWKWPWWPWRR-NH2 (13aa)	Other structure	4	2–64	[89,106,133]
KS-12		KRIVQRIKDFLR (12aa)	AH	256	64–256	[137]
KR-20	*Homo sapiens*	KRIVQRIKDFLRNLVPRTES (20aa)		64	16–32	
KR-30		KSKEKIGKEFKRIVQRIKDFLRNLV PRTES (30aa)		16	8–16	
Lactoferrin (Lf)	Camel (Colostrum milk)	Large protein	complex	—	Significant clearance of *A. baumannii*	[138]
Lactoperoxidase (Lpo)					rates in lung	
Latarcin 2a	*Pleuronectes americanos* (Winter flounder)	H-GLFGKLIKKFGRKAISYA VKKARGKH-OH (26aa)	AH	16	8–64	[93]
LL-37	*Homo sapiens*	LLGDFFRKSKEKIGKEFKRIVQRIK DFLRNLVPRTES (37aa)	AH	32	16–32	[137,139]
LS-AMP-E1	*Lycosa sinensis* (Chinese wolf spider)	AGMKNIIDAIKKKLGGKL (18aa)	AH	—	25–100 *	[140]
LS-AMP-F1		TGLGKIGYLMKKLLSKAKV (19aa)	AH	—	3.1–12.5 *	[141]
LS-sarcotoxin	*Lucilla serricata*	GWLKKIGKKIERVGQHTRDATIQTIGVAQQAANVAATLK-NH2 (39aa)	AH	4	4–8	[141]
LS-stomoxyn		GFRKRFNKLVKKVKHTIKETANVSKDVAIVAGSGVAVGAAM-NH2 (41aa)	AH	8	4–16	
Lynronne-1	Bovine rumen microbiome	LPRRNRWSKIWKKVVTVFS (19aa)	AH	4	—	[142]
Magainin-1	Frog skin peptide	GIGKFLHSAGKFGKAFVGEIMKS (23aa)	AH	—	256	[100,101]
Magainin-2	Frog skin peptide	GIGKFLHSAKKFGKAFVGEIMNS (23aa)	AH	9.8–64	4.9–64	[100,101,143]
Mastoparan	*Vespula lewisii* (Hornet venom)	INLKALAALAKKIL (14aa)	AH	4	—	[106,144,145]
Mastoparan-AF (EMP-AF)	*Vespa affinis* (Hornet venom)	INLKAIAALAKKLF-NH2 (14aa)	AH	2–16	2–16	[146]
Mastoparan-Chitosan Nanoconstruct	*Vespula lewissi* (Wasp venom)	INLKALAALAKKIL-NH2 (14aa)	AH	—	2–4	[147]
Maximin H2	*Oreochromis niloticus* (Nile Tilapia)	ILGPVLSMVGSALGGLIKKI-NH2 (20aa)	AH	64	16–128	[93]
Mdc	*Housefly larvae*	GWLKKIGKKIERVGQHTRDATIQ TIGVAQQANAVAATLKG (40aa)	D-helix	4	4	[148]
Melittin	*Apis mellifera* (European honeybee)	GIGAVLKVLTTGLPALISWIKRKRQQ (26aa)	AH	0.25–4	0.25–25	[106,149,150]
Melittin with colistin (COL)	*Apis mellifera* (European honeybee)	GIGAVLKVLTTGLPALISWIKRKR QQ (26aa) + COL	AH	0.37–0.5	0.19–0.37	[151]
Melittin with imipenem (IPM)		GIGAVLKVLTTGLPALISWIKRKR QQ (26aa) + IPM	AH	0.31–0.37	0.12–0.25	
Mini-ChBac7.5 Nα	*Capra hircus* (Domestic goat)	RRLRPRRPRLPRPRPRPRPRPR (22aa)	AH	—	2 *	[152]
Mini-ChBac7.5 Nβ		RRLRPRRPRLPRPRPRPRPRP (21aa)	AH	—	4 *	
Myxinidin 2	*Myxine glutinosa L* (Atlantic hagfish)	KIKWILKYWKWS (12aa)	AH	—	12.5	[153]
Myxinidin 3		RIRWILRYWRWS (12aa)	BS	—	6.3	
N10	Blood biopanning	ACKDVNTSMCGGK (13aa)	AH	500	500	[154]
NA-CATH	*Naja atra* (Snake venom)	KRFKKFFKKLKNSVKKRAKKFFKK PKVIGVTFPF (34aa)	AH	10	10	[85]
NB2	Biofilm biopanning	ACERSIRTVCGGK (13aa)	AH	500	500	[154]
NDBP5.8	*Opisthacanthus cayaporum* (Scorpion venoms)	GILGKIWEGVKSLI (14aa)	AH	>25	>25	[81]
Nisin	*Lactococcus lactis* (Probiotic bacterium)	MSTKDFNLDLVSVSKKDSGASPRITSISLCTPGGKTGALNGCNMKTATCHCSIHVSK (34aa)	NF	128	64–128	[155]
Nisin + P10 combined	*Lactococcus lactis* (Probiotic bacterium) + Synthetic derivated	MSTKDFNLDLVSVSKKDSGASPRITSISLCTPGGKTGALNGCNMKTATCHCSIHVSK (34aa) + LAREYKKIVEKLKRWLRQVLRTLR (24aa)	NF	32	16–32	
Nodule-specific cysteine-rich (NCR) peptide and its derivatives	Medicago trunculata	RNGCIVDPRCPYQQCRRPLYCRRR (24aa)	AH	1.6–25 MBC	—	[156,157]
NRC12	Flatfish Genes	GWKKWFNRAKKVGKTVGGLAVDHYL-NH2 (25aa)	AH	16	8–32	[93]
Nuripep 1653	Derived from the P54 nutrient reservoir protein (aa 271–292) pea protein from Pisum sativum	VRGLAPKKSLWPFGGPFKSPFN (22aa)	AH	—	12	[158]
OH-CATH30	King cobra (Snake venom)	KFFKKLKNSVKKRAKKFFKKPRVI GVSIPF(30aa)	AH	10	10	[122]
Pexiganan	Frog skin peptide	GIGKFLKKAKKFGKAFVKILKK (22aa)	AH	1–8	1–8	[88,159,160]
PGLa-AM1	Frog skin peptide	GMASKAGSVLGKVAKVALKAAL-NH2 (22aa)	AH	16–128	16–128	[96,161]
Pilosulin	Ant venom (toxin pilosulin)	GLGSVFGRLARILGRVIPKV-NH2 (20aa)	AH	16	8–16	[93]
Pleurocidin	*Pleuronectes americanus* (Winter flounder)	GWGSFFKKAAHVGKHVGKAALTHYL-NH2 (25aa)	AH	16	8–32	[93]
Polydin-I	*Polybia dimorpha* (Social wasp)	AVAGEKLWLLPHLLKMLLTPTP (22aa)	AH	>25	>25	[81]
Polybia-MPII	*Pseudopolybia vespiceps* testacea	INWLKLGKMVIDAL (14aa)	AH	12.5	25	[81]
Protegrin-1	*Cimex lectularius*	RGGRLCYCRRRFCVCVGR-NH2 (18aa)	AH	—	2–8	[162]
P307SQ-8C	Hepatitis B virus	NAKDYKGAAAEFPKWNKAGGRV LAGLVKRRKSQSRESQC (39aa)	NF	125	62.5–125	[163]
Ranalexin	*Rana catesbeiana* (American bulfrog)	LGGLIKIVPAMICAVTKKC (19aa)	AH	—	4–18	[115]
SAAP-148	*Homo sapiens*	LKRVWKRVFKLLKRYWRQLKKPVR (24aa)	AH	—	6	[164]
Spiniferin	*Heterometrus petersii* (Scorpion venom gland)	ILGEIWKGIKDIL (13aa)	AH	>40	—	[94]
[S7K, G11K] Alytesirrin-2a	Frog skin peptide	ILGKLLKTAAKLLSNL-NH2 (16aa)	AH	—	8	[165]
SMAP29	Sheep myeloid	RGLRRLGRKIAHGVKKYGPTVLRIIRIAG (29aa)	AH	8	4–32	[93]
Tachyplesin III	*Tachypleus gigas* and *Carcinoscorpius rotundicauda* (Horseshoe crabs)	KWCFRVCYRGICYRKCR-NH2 (17aa)	BS 2 dissulfite bridges	—	8–16	[120]
Temporin A	*Rana temporaria* (European red frog)	FLPLIGRVLSGIL-NH2 (13aa)	AH	128	—	[88]
TP4	*Oreochromis niloticus* (Nile tilapia)	FIHHIIGGLFSAGKAIHRLIRRRRR (25aa)	AH	16	8–32	[93]
Venon cocktail proteins	*Leiurus quinquestriatus* (Scorpion venom)	Cocktail	NF	—	50.6% of inhibition (20 mg/mL of venom)	[166]
VsCT1	*Heterometrus petersii* (Scorpion venom gland)	FLKGIIDTVSNWL (13aa)	AH	>40	—	[94]
WAM-1	*Macropus eugenii* (Tammar wallaby)	KRGFGKKLRKRLKKFRNSIK KRLKNFNVVIPIPLPG (36aa)	AH	8.12	4–64	[89,167]
WLBU2- arginine-rich amphiphilic peptide	Skin wounds	RRWVRRVRRWVRRVVRVVRRWVRR (24aa)	NF	~7.484	~7.484	[168]
ZY4 cathelicidin-BF-15 derived	*Bungarus fasciatus* (Snake venom)	VCKRWKKWKR KWKKWCV-NH2 (17aa)	Cyclic SH-bridge	—	4.6–9.4	[169]

AH, α-helical; BS, β-sheet; *, result in µM; aa, amino acid; ~, mean of; NF, not found; NPs, nanoparticles; ATBS, antibiotic-susceptible; Ref., reference.

**Table 4:** Synthetic AMPs point out anti-*A. baumannii* activity.

Peptide	Source	Sequence (nº Amino Acid)	Structure	MIC against *A. baumannii* (μg/mL)		Ref.
				ATBS	MDR	
AS-CATH8	Synthetic peptide	KRVNWAKVGRTALKLLPYIFG (21aa)	AH	0.6	—	[86]
BmKn2-7		FIKRIARLLRKIF-NH2 (13aa)	AH	5	5–10	[95]
BmKn2-7R	Synthetic peptide	FIRRIARLLRRIF-NH2 (13aa)	AH	2.5	2.5–5	
BmKn2-7K		FIKKIAKLLKKIF-NH2 (13aa)	AH	2.5	2.5–5	
BP100		KKLFKKILKYL (11aa)	AH	—	4	[170]
BP214	Hybrid peptide	KKLFKKILRYL (11aa)	AH	2	—	
BP214 analogs		KKLFKKILRYL (11aa) analogs	AH	>64	—	
CA(1–8)-ME(1–12) (CAME)	Chimeric peptide	KWKLFKKIGIGAVLKVLTTG-NH_2_ (20aa)	AH	3.12	3.12–12.5	[171]
CA(1–8)-MA(1–12) (CAMA)		KWKLFKKIGIGKFLHSAKKF-NH_2_ (20aa)	AH	12.5	3.12–12.5	
Cecropin-4	Synthetic peptide	GWLKKIGKKIERVGQNTRDATIQ AIGVAQQAANVAATLKG (40aa)	AH	4	*4*	[172,173]
Cecropin A (1–8) melittin (1–10) (CAME)	Hybrid peptide	KWKLFKKIGIGAVLKVLTTG-NH2 (20aa)	AH	32	8–32	[93]
Ceragenins; CSA-192; CSA-131; D-150-177C; HBcARDderivative	Cholic acid synthetic mimics	Steroids compounds	NF	—	—	[174]
Chex1-Arg20 amide (ARV-1502)	NA	RPNKPRPYLPRPRPPRPVR-NH2 (19aa)	NF	—	Reduction of bacterial load	[175]
D-AP19	Hybrid peptide	RLFRRVKKVAGKIAKRIWK-NH2 (19aa)	NF	7.81	3.91–15.63	[176]
DGL 13K	Synthetic derived D-enantiomers of GL13K derived from the salivary protein BPIFA2	GKIIKLKASLKLL-NH2 (13aa)	NF	—	8–32	[177]
D-Mt6	Synthetic peptide	KFKKTAKWLIKSAWLLLKSLALKMK (25aa)	AH	8	—	[178]
DP7	Computationally designed	VQWRIRVAVIRK (12aa)	AH	—	4–16	[179,180,181]
ECPep-D	Synthetic peptide	RPFTRAQWFAIQHISPRTIAMRAINNYRWR (30aa)	NF	37.57	—	[182]
ECPep-2D-Orn	Synthetic peptide	OPFTOAQWFAIQHISPOTIAMOAINNYOWO (30aa)	NF	17.53	—	[182]
GW-A2		GAKYAKIIYNYLKKIANALW (20aa)	AH	32	8–32	[93]
GW-H1a	Synthetic peptide	GYNYAKKLANLAKKFANALW-NH2 (20aa)	AH	32	8–32	
GW-Q6		GIKIAKKAITIAKKIAKIYW (20aa)	AH	16	8–16	
HP(2–9)-MA(1–12) (HPMA)	Chimeric peptide	AKKVFKRLGIGKFLHSAKKF-NH_2_ (20aa)	AH	6.25	3.12–6.25	[171]
HP(2–9)-ME(1–12) (HPME)		AKKVFKRLGIGAVLKVLTTG (20aa)	AH	6.25	3.12–12.5	
I16K-piscidin-1 and analogs	Hybrid striped bass *Morone saxatilis x M. chrysops*	FFHHIFRGIVHVGKTIHRLVTG (22aa)	NF	—	3.1	[183]
IKR18	Computationally designed	IKRQYKRFFKLFKWFLKK (18aa)	AH	1	—	[184]
LJ-hep2_(66–86)_	Synthetic peptide	IKCKFCCGCCTPGVCGVCCRF (21aa)	NF	—	1.5–3	[185]
LyeTx I-bPEG	Synthetic peptide	WLTALKFLGKNLGKLAKQQCAKL (PEG) (24aa)	AH	—	—	[186]
mCM11, cecropin–melittin 11	Synthetic peptide	NH2-WRLFRRILRVL-NH2 (11aa)	AH	32	<4–>512	[187]
MSI-78	Synthetic peptide, magainin analog	GIGLPLLLALLPGLAPVLILLL-NH2 (22aa)	AH	—	5	[188]
Mt6	Synthetic peptide	KKFKKTAKWLIKSAWLLLKSLALKMK (26aa)	AH	8	—	[178]
NCR169C and its substitution derivatives	*Synthetic peptide*	KSKKPLFKIWKCVENVCVLWYK	AH	1.6–12.5 MBC	—	[189]
Octominin, Octominin-CNPs	Synthetic derived, defensin 3 of *Octopus minor*	GWLIRGAIHAGKAIHGLIHRRRH (23aa)	AH	—	5	[190,191]
Octopromycin	Synthetic peptide	N-RRLIRTDTGPIIYDYFKDQLLKKGMVI LRESMKNLKGM-C (38aa)	AH	—	50	[192]
OG1410	ApoE-based synthetic peptide	acetyl-ASAib-LRKL-Aib-KRLL-amide	AH	16	16	[193]
Omega 76-shuft1	Computationally designed	AFLLKKKKGIIFFEKAKKGK (20aa)	AH	—	4–16	[194]
Omiganan	Synthetic peptide	ILRWPWWPWRRK-NH2 (12aa)	AH	32	—	[88]
r-Omiganan		KRRWPWWPWRLI-NH2 (12aa)	AH	16	—	
OMN6	Synthetic peptide	H-M-C-KWKLFKKIEKVGQNIRDGIIKA-GP-AVAVVGQATQIAK-C-NH2 (40aa)	AH	8	4–8	[195,196]
′Ω17 family peptides	Computationally designed	RKKAIKLVKKLVKKLKKALK (20aa)	AH	2	1–8	[194]
′Ω76 family peptides		FLKAIKKFGKEFKKIGAKLK (20aa)	AH	4	2–8	
P10	Synthetic derivated	LAREYKKIVEKLKRWLRQVLRTLR (24aa)	NF	4	8–32	[155]
P10 + Nisin combined	Synthetic derivated + *Lactococcus lactis* (Probiotic bacterium)	LAREYKKIVEKLKRWLRQVLRTLR (24aa) + MSTKDFNLDLVSVSKKDSGASPRITSISLCTPGGKTGALNGCNMKTATCHCSIHVSK (34aa)	NF	1	4–16	
PapMA	Hybrid peptide	RWKIFKKIPKFLHSAKKF-NH2 (18aa)	AH	32	16–32	[197]
pepD2	Computationally designed	WKKLKKLLKKLKKL-NH2 (14aa)	AH	8	-	[198]
PLP-3	Synthetic peptides derived from the innate immune system of vertebrates	~RRPVCVVPLPRVPCLRRR~	B- hairpin	1–2	1–2	[199]
PNA (RXR)4 XB	Peptide nucleic acid conjugated to (RXR)4 Phosphorodiamidate Morpholino Oligomers	RXRRXRRXRRXRXB (14aa)	NF	—	1.25 *	[200]
Pro9-3	Computationally designed	RLWLAIWRR-NH2 (9aa)	AH	16	8–64	[201]
Pro9-3D		RLWLAIWRR-NH2 (9aa)	AH	8	4–16	
RR	Computationally designed	WLRRIKAWLRR (11aa)	AH	—	25–99	[202,203]
RR2		WIRRIKKWIRRVHK (14aa)	AH	—	3–6	
RR-4		WLRRIKAWLRRIKA (14aa)	AH	—	3–6	
R-Pro9-3	Computationally designed	RRWIALWLR-NH2 (9aa)	AH	16	8–32	[201]
R-Pro9-3D		RRWIALWLR-NH2 (9aa)	AH	8	4–16	
S4A	NA	IOWAGOLFOLFO-NH2 (12aa)	AH	100	50	[204]
SAAP-148 NPs	*Synthetic peptide*	LKRVWKRVFKLLKRYWRQLKKPVR (24aa) + NPs	AH	—	—	[205]
Scolopendin A2	Synthetic peptide	AGLQFKVGRIGRLLRK (16aa)	NF	—	16	[206]
SPO	NA	NINONWNANGNONLNFNONLNFNO-NH2 (22aa)	AH	100	50	[204]
Stapled AMP Mag (i + 4)1, 15(A9 K, B21A, N22 K, S23 K)	NA, based on magainin two structure	Mag(i + 4)1,15(A9K,B21A,N22K,S23K)	complex	—	—	[207]
TAT-RasGAP_317–326_ anticancer peptide	Chimeric (cell penetrating sequence + Src homology sequence)	G48RKKRRQRRR^57^ + W^317^MWVTNLRTD^326^	AH	Growth inhibitory effect	—	[208,209]
TAT-RasGAP_317–326_	Chimeric peptide	G48RKKRRQRRR57 (10aa)	NF	—	—	[210]
TP2-5	Computationally designed	KKCIAKAILKKAKKLLKKLVNP (22aa)	AH	3.125	1.56–3.125	[211]
TP2-6		KKCIAKAILKKAKKLLKDLVNP (22aa)	AH	3.125	3.125–12.5	
Trichogin analogs	Synthetic peptide	1-Oct-Aib-Gly-Leu-Aib-Gly-Gly-Leu-Aib-Gly-Ile-Lol		>128	—	[212]
zp3	Synthetic peptide	GIIAGIIIKIKK-NH2 (12aa)	AH	4	—	[213]

AH, α-helical; BS, β-sheet; NA, unavailable; *, result in µM; aa, amino acid; >, bigger then; <, less than; NF, not found; NPs, nanoparticles; Ref., reference.

**Table 5:** Gram-negative bacterial resistance mechanisms against AMP.

Mechanism	Gram-Negative Bacteria	Reference
Degradation or sequestration by secreted proteins	Proteolytic degradation	[314,315,316]
Impedance by exopolymers or biofilm matrix molecules	Alginate, polysialic acid	[304,317,318]
Cytoplasmic outer membrane alteration	Increased IM rigidity by PG acylation	[319]
Surface modification	Repulsion by lipid A phosphate modification increased OM rigidity by lipid A acylation. O-antigen of LPS	[320,321]
Multidrug efflux pump	Export via efflux pumps (RND family)	[322,323]

## Data Availability

The data presented in this study are available on request from the corresponding author.
